# MAGa: Monoclonal Autoimmune Gammopathies

**DOI:** 10.3390/cancers18111770

**Published:** 2026-05-28

**Authors:** Stephanie Torres, Sarah E. Wheeler, Michael R. Shurin

**Affiliations:** 1 Department of Pathology, University of Pittsburgh and University of Pittsburgh Medical Center, Pittsburgh, PA 15213, USA; torress6@upmc.edu (S.T.); wheelerse3@upmc.edu (S.E.W.); 2 Clinical Immunopathology, University of Pittsburgh Medical Center, 3477 Euler Way, Clinical Laboratory Building, Room 4024, Pittsburgh, PA 15213, USA

**Keywords:** plasma cell disorders, autoantibody, autoinflammation, monoclonal gammopathy

## Abstract

There is a growing recognition of bidirectional relationships between immune phenomena in plasma cell disorders, such as multiple myeloma and its precursor malignancies, including autoimmune phenomena that either precede or complicate the development of neoplasms. The development of specific autoimmune disorders is a well-known aspect of B-cell lymphoproliferative diseases. At the same time, the development of plasma cell dyscrasia in patients with autoimmune diseases is well established. This may suggest that some subclones of multiple myeloma and its precursor, monoclonal gammopathy of undetermined significance, originate in the setting of persistent autoimmune activation. Interestingly, monoclonal immunoglobulins, which are key biomarkers for characterizing and monitoring monoclonal gammopathies, have long been overlooked in clinical research because they are thought to have no significant antibody function. It is plausible that unusual clinical consequences may occur when monoclonal myeloma paraproteins bind to endogenous self-antigens. Published clinical data illustrate this possibility well. This review synthesizes published evidence demonstrating that many patients with multiple myeloma and related plasma cell dyscrasias exhibit specific clinical manifestations of antigen–antibody interactions between a monoclonal immunoglobulin produced by proliferating B-cell or plasma-cell clones and autologous antigens, including neural antigens, insulin, complement components, and others. We present pilot data showing antinuclear antibody reactivity in 38% of IgM monoclonal gammopathy sera. Numerous experimental and clinical data support the importance of identifying the target of the monoclonal immunoglobulins in monoclonal gammopathy of undetermined significance, smoldering multiple myeloma, and multiple myeloma. This approach not only aids in selecting an appropriate treatment strategy and predicting prognosis but may also guide the development of targeted therapies for managing monoclonal gammopathy cases that present with a paraprotein reactive against a known self-antigen. We propose the term monoclonal autoimmune gammopathies (MAGa) to describe this clinically distinct subset of plasma cell disorders characterized by pathogenic autoantibody activity of the monoclonal immunoglobulin.

## Simple Summary

Monoclonal autoimmune gammopathies are a group of conditions in which abnormal immune proteins produced by clonal B cells or plasma cells are autoreactive and cause organ or tissue damage in addition to the ongoing premalignant or malignant processing. While monoclonal proteins are commonly detected in older adults and often remain harmless, they can sometimes trigger autoimmune diseases affecting multiple organs, including the kidneys, nerves, skin, and blood vessels. This review aims to establish a comprehensive framework for understanding these monoclonal autoantibody conditions, particularly monoclonal gammopathies of unknown significance, by clarifying how to identify them early, distinguish them from benign findings, and guide treatment decisions. The findings will help the research community recognize these monoclonal autoimmune disorders as distinct clinical entities requiring multidisciplinary care, potentially improving outcomes through earlier detection and targeted therapies that address both the underlying clonal process and the resulting autoantibody-mediated organ damage.

## 1. Introduction

There is a growing recognition of immune phenomena in hematologic malignancies, including autoimmune disorders that either precede or complicate the development of neoplasms. Hematologic conditions, like cytopenias, and non-hematologic conditions, such as polyneuropathies, in autoimmune disorders have been linked to lymphoproliferative diseases, including multiple myeloma, chronic lymphocytic leukemia (CLL), chronic myelomonocytic leukemia (CMML), lymphomas, myelodysplastic syndromes (MDS), acute leukemias, and myeloproliferative neoplasms [1,2,3]. The risk of B-cell non-Hodgkin lymphoma is also notably higher in patients diagnosed with autoimmune diseases [4,5]. Autoimmune hemolytic anemia and immune thrombocytopenia frequently complicate both lymphoid and myeloid neoplasms [6,7]. Dasanu et al. reported that nearly 50% of patients with marginal zone lymphoma (MZL) had autoimmune conditions, including autoimmune hemolytic anemia, immune thrombocytopenia, Hashimoto thyroiditis, rheumatoid arthritis, psoriasis, and systemic lupus erythematosus (SLE) [8]. Diffuse large B cell lymphoma may be associated with Hashimoto thyroiditis in 31% of cases and rheumatoid arthritis in 23% of cases [9].

Autoimmune cytopenias are observed in up to 10% of patients with CLL and occur more frequently in specific cases of non-Hodgkin lymphoma, while they are present in less than 1% of patients with low-risk MDS and CMML. Autoimmune diseases are reported in about 30% of patients with myeloid and lymphoid malignancies and include, but are not limited to, immune hemostatic disorders such as thrombotic thrombocytopenic purpura, acquired hemophilia, and antiphospholipid syndrome, which can be severe and fatal [4]. The link between autoimmune diseases and many B-cell neoplasms has led to the hypothesis that the evolving origin of B cells may underlie their malignant response to prolonged autoimmune stimuli [3]. Validation of this hypothesis showed that predominantly germinal center-derived B-cell neoplasms had numerous associations with autoimmune diseases: diffuse large B-cell lymphoma, follicular lymphoma, and Hodgkin lymphoma were linked to 15, 7, and 5 autoimmune diseases, respectively, and shared significant associations with five diseases (rheumatoid arthritis, Sjogren syndrome, polymyositis/dermatomyositis, SLE, and immune thrombocytopenic purpura) [3]. Conversely, predominantly non-germinal center neoplasms, such as acute lymphocytic leukemia, CLL, and mantle cell lymphoma, showed associations with only 1–2 autoimmune conditions. Furthermore, when autoantibodies are present in most (90%) CLL cases, they are polyclonal, high-affinity IgG produced by non-malignant self-reactive B cells rather than by the malignant clone [9].

**Figure 1:**
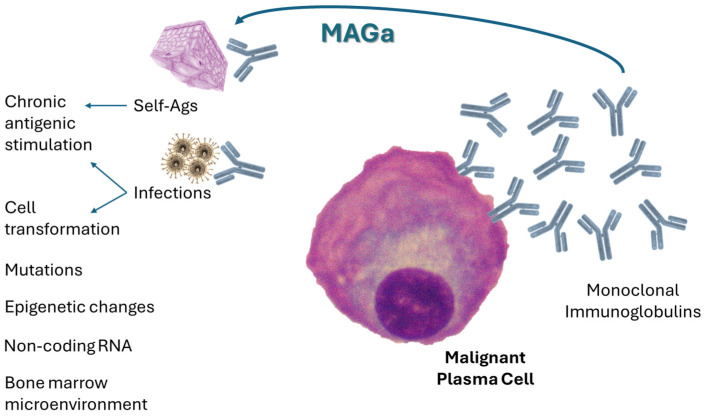
**The mechanisms underlying the possible transformation process of a normal plasma cell to the cancerous phenotype in monoclonal gammopathies, including MAGa**. Monoclonal gammopathies are blood malignancies characterized by the expansion of a plasmacytic clone that produces monoclonal immunoglobulin. While the mechanisms behind the malignant transformation of plasma cells mainly involve DNA mutations, the significant role of epigenetic changes is also well recognized. Antigenic stimulation can induce subgroups of plasma cell dyscrasias, including MGUS and multiple myeloma. This primary antigenic stimulus can be identified by pinpointing the specific targets of monoclonal immunoglobulins from patients’ sera. Many cases of MGUS and multiple myeloma have been linked to recognition of antigenic determinants on HBV, HCV, EBV, CMV, enteroviruses, *H. pylori*, and autoantigens. Therefore, chronic stimulation by self-antigens or infectious antigens might promote the pathogenetic pathways of some subsets of MGUS and myeloma. In monoclonal autoimmune gammopathies (MAGa), repeated exposure to autoantigens may contribute to the development of multiple myeloma. Alternatively, these associations could reflect an underlying immune disorder that exists years before the diagnosis of multiple myeloma, thereby illustrating the natural course of the disease.

Multiple myeloma, a tumor of germinal center-experienced plasma cells, has a strong association with autoimmune conditions. In most monoclonal gammopathies, the clinical picture may be further complicated by the ability of unusual paraproteins to recognize certain host antigens (Figure 1). Monoclonal immunoglobulin, as a characteristic of essential monoclonal gammopathy, may target a specific self-antigen, resulting in functional or structural consequences and a detectable clinical syndrome. It is unclear whether the monoclonal immunoglobulin with sufficient affinity for an epitope on a normal host protein appeared by chance. The view that such events are probably random, simply incidental, and caused by a malignant clone of B or plasma cells producing a monoclonal antibody that mimics an autoantibody is attractive, but it cannot explain the non-random distribution of identified autoimmune targets.

Alternatively, modern modifications and adaptations of the “*horror autotoxicus*” and ‘forbidden clone’ conceptions might be useful for explaining the appearance of malignant B and plasma cell clones from pre-existing hyperactivated autoimmune B cells [10,11]. Limited preclinical studies have reported somatic mutations in immunoglobulin genes in Waldenström’s macroglobulinemia, indicating a role for antigenic stimulation in the development of this monoclonal gammopathy [12,13]. However, numerous tolerogenic and checkpoint signaling pathways should prevent autoantigen-stimulated persistent progression of self-reactive B and T cells. Several genes that encode regulators of these pathways overlap with those that control tumor suppression. It is speculated that congenital flaws in genes encoding tolerance regulators may trigger autoimmunity stochastically, as suggested for an inherited susceptibility to malignant transformation [14]. Various hereditary and somatic mutations must accumulate to provide an opportunity for an autoreactive cell clone to bypass tolerance barriers, leading to the development of autoimmune conditions [14].

Regardless of the cause of monoclonal autoimmune gammopathies (MAGa), the presence of monoclonal immunoglobulins that target autoantigens and bypass tolerogenic blockade is an intriguing fundamental immunological issue and an important clinical immunological and immunopathological challenge.

## 2. Monoclonal Gammopathies

Monoclonal proteins are not uncommon, with a prevalence in the United States of about 5%, and their incidence increases with age [15]. Although most patients are asymptomatic, most cases are caused by a clonal plasma cell disorder. Monoclonal gammopathy refers to the presence of a monoclonal immunoglobulin (M-protein) or a monoclonal free light chain (FLC) in plasma, serum, CSF, urine, or both that is produced by clonal plasma cells or B lymphocytes. The variable regions of the monoclonal immunoglobulin are explicit to the original malignant clone since they are the result of gene rearrangements and mutations. Monoclonal gammopathy can indicate multiple myeloma, immunoglobulin light-chain amyloidosis, or other lymphoid malignant diseases [15]. Monoclonal gammopathy of undetermined significance (MGUS) and smoldering multiple myeloma are clinically asymptomatic premalignant conditions with variable risk of progression to multiple myeloma. MGUS is confirmed when end-organ damage or clinical symptoms are absent, and only a small amount of M-protein and a low volume of bone marrow plasma cells are present [16]. Rarely, monoclonal gammopathy can cause symptoms in the absence of malignant disease, which is called monoclonal gammopathy of clinical significance (MGCS). MGCS represents symptomatic monoclonal gammopathies, which, however, are outside of the diagnostic criteria for cancerous plasma cell pathology [17].

Among plasma cell disorders, or plasma cell dyscrasias (PCD), multiple myeloma and its precursor stages are among the best understood. Multiple myeloma, the second most common hematological cancer, represents the final stage in a spectrum of plasma cell dyscrasias characterized by the clonal growth of abnormal plasma cells in the bone marrow. It is consistently preceded by the premalignant condition MGUS. MGUS, affecting more than 4–5% of adults over 50 years old, is considered a necessary precursor to several lymphoplasmacytic cancers, including immunoglobulin light-chain amyloidosis, multiple myeloma, and Waldenström macroglobulinemia. Smoldering multiple myeloma is the disease representing the transitional phase between asymptomatic MGUS and active multiple myeloma [15]. The progression of MGUS and smoldering multiple myeloma to clinically defined multiple myeloma involves specific genomic and microenvironmental events; however, it is generally unclear whether these events are causes or effects of disease progression [18]. Additionally, multiple myeloma can progress to an extramedullary stage, with certain cases advancing to plasma cell leukemia.

Waldenström macroglobulinemia is a low-grade non-Hodgkin lymphoma characterized by bone marrow lymphoplasmacytic infiltrates and hypersecretion of monoclonal IgM immunoglobulins. According to the WHO classification, Waldenström macroglobulinemia corresponds to lymphoplasmacytic lymphoma (LPL), and the MYD88 L265P mutation is observed in over 90% of cases [19]. Somatic hypermutation supports the view that the Waldenström macroglobulinemia B-cell clone in most patients originates before the germinal center stage. Therefore, elevated levels of IgM are also detected in IgM MGUS and smoldering Waldenström macroglobulinemia, forming the group of IgM-associated pre-neoplastic diseases and neoplasms.

There are also several known complex clinical syndromes with a proven association with detectable monoclonal immunoglobulins. For instance, Schnitzler syndrome is a rare autoinflammatory disease characterized by the presence of a monoclonal protein: IgM-κ in >85% of cases and IgM-λ or IgG-κ in a minority of cases [20]. Evolution of this clonal disorder to LPL, Waldenström macroglobulinemia, or IgM myeloma has been reported in 15–20% of patients [21]. POEMS syndrome (also known as Crow–Fukase syndrome or Takatsuki disease) is considered to be a paraneoplastic syndrome due to an underlying plasma cell malignancy, and is characterized by polyneuropathy, organomegaly, endocrinopathy, monoclonal plasma cell disorder, and skin changes. This disease is usually an IgG-λ or IgA-λ subtype and differs from other paraproteinemic abnormalities by its potential multiorgan involvement. Monoclonal immunoglobulins produced by monoclonal plasma cells are a fundamental diagnostic element for POEMS syndrome [22]. TEMPI syndrome, with its five characteristics (telangiectasias, elevated erythropoietin and erythrocytosis, monoclonal gammopathy, perinephric fluid collections, and intrapulmonary shunting) is also associated with detectable monoclonal immunoglobulins [23,24]. The presence of an IgG-κ paraprotein in evaluated cases may suggest a pathogenic role of the paraprotein and suggest that TEMPI cases may belong to MGCS. This group may also include CANOMAD syndrome, clinically confirmed by ophthalmoplegia, cold agglutinins, chronic ataxic neuropathy, monoclonal IgM immunoglobulins, and disialosyl antibodies [25,26].

Monoclonal gammopathy-associated systemic capillary-leak syndrome, known as Clarkson’s disease, is a condition with recurrent episodes of capillary hyperpermeability in the context of a monoclonal gammopathy [27]. Production of monoclonal IgG-κ and IgA-κ has been described in this rare condition. Characterized largely by hypereosinophilia and angioedema, Gleich syndrome is another rare disorder associated with increased levels of monoclonal or polyclonal IgM [28]. Castleman disease is also a group of disorders characterized by lymphoproliferative abnormalities, such as MGUS. In some cases, such as plasma cell type Castleman disease, patients present with monoclonal gammopathy mimicking myeloma [29]. Multicentric Castleman disease can also be found with paraneoplastic syndrome, such as POEMS syndrome.

**Figure 2:**
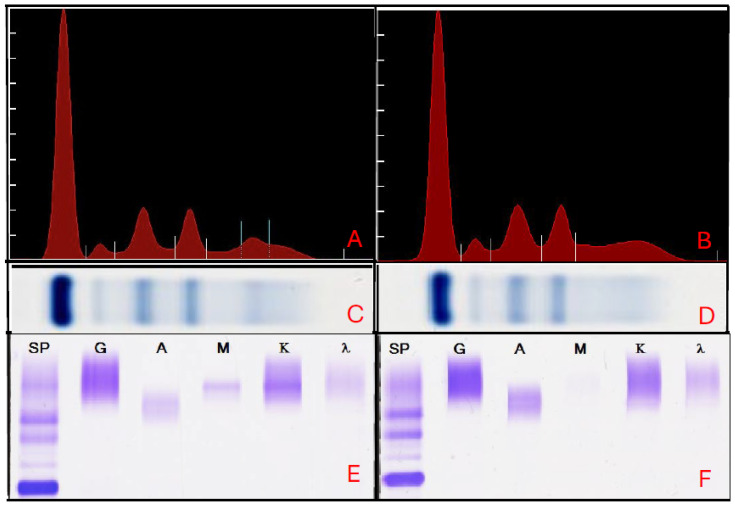
**Example of positive and negative results for monoclonal gammopathy using serum protein electrophoresis and immunofixation electrophoresis.** Serum samples were collected in separator tubes, allowed to clot at room temperature, and then centrifuged before analysis. The samples were examined for monoclonal gammopathy using agarose gel serum protein electrophoresis (SPEP) and serum immunofixation electrophoresis (IFE). Total protein concentration was measured by refractometry, and all samples were applied to agarose gels for electrophoresis using Applicator Blades, following the manufacturer’s instructions (Helena Laboratories, Beaumont, TX, USA). Electrophoresis was performed under standardized voltage and migration conditions according to the manufacturer’s instructions, followed by protein fixation and staining for visualization on a SPIFE 3000 (Helena Laboratories), as described earlier [37]. SPEP results show a monoclonal M spike in the gamma region in positive cases, as demonstrated by densitometric evaluation (**A**) of an electropherogram (**C**), whereas negative cases display a smooth, polyclonal protein distribution (**B**,**D**). The M Spike values in our sampled population ranged from 0.25 to 4.41 g/dL, with both the mean and median of 0.42 g/dL. For IFE, proteins were stained with monospecific antisera against IgG, IgA, and IgM heavy chains, as well as kappa and lambda light chains, after electrophoresis. The samples were then incubated, washed to remove unbound proteins, and stained to detect immune complexes. Positive IFE results show discrete bands corresponding to specific IgM kappa monoclonal immunoglobulins (**E**), while negative results lack such bands, indicating no monoclonal gammopathy in most cases (**F**). All procedures followed standard laboratory protocols for detecting and characterizing monoclonal proteins at the College of American Pathologists-accredited, CLIA-certified UPMC Clinical Immunopathology Laboratory. Results of one independent representative experiment are shown in each panel.

In most cases of PCD, the presence of monoclonal immunoglobulins is a key diagnostic characteristic. The guidelines of the International Myeloma Working Group and the College of American Pathologists advise running serum total protein electrophoresis and serum free light chain analysis as a first-line evaluation, followed by immunofixation for follow-up. The monoclonal immunoglobulin band, or M-protein, also known as an M-spike, is commonly detected by serum or urine protein electrophoresis, with further characterization of monoclonal immunoglobulin provided by immunoelectrophoresis. An example of these electrophoresis results is shown in Figure 2. The most frequent M-protein isotype is IgG (50–60%), followed by IgA (15–25%), IgM (10–15%), IgD and IgE (<3–5%), biclonal (<1%), and no measurable monoclonal protein (1–3%) [30].

Remarkably, monoclonal immunoglobulins, which are major biomarkers for characterizing and monitoring clonal gammopathies, have long been overlooked in clinical research because they are considered to have no significant antibody function. At the same time, potential recognition of various proteins and microbial antigens by paraproteins in individual clinical cases was recognized several decades ago (Figure 1), as predicted by Jan Waldenström [31,32]. Some of those results were confirmed later (Table 1). For instance, purified monoclonal antibodies from patients with MGUS and multiple myeloma who had previously been infected with the hepatitis C virus (HCV) “reacted against HCV” in 6 out of 9 patients [33]. The fact that some of these patients demonstrated better evolution of plasma cell dyscrasia after antiviral treatment allowed the authors to suggest a causal relationship between HCV infection and progression of gammopathy [33]. The authors confirmed their findings by demonstrating the specific recognition of hepatitis B virus (HBV) antigens HBx and HBc by monoclonal immunoglobulins in HBV-infected patients with monoclonal gammopathy [34]. Furthermore, patients presenting with monoclonal immunoglobulins directed against the virus in the context of HCV infection may have an increased risk of plasma-cell malignancy [35]. Harb et al. demonstrated the specificity of some monoclonal immunoglobulins from MGUS patients to poliovirus and coxsackievirus antigens and concluded that virus-initiated MGUS are not rare, and some may evolve toward myeloma [36].

**Table 1:** Examples of reported monoclonal autoreactive antibodies in patients with plasma-cell dyscrasias.

Monoclonal Gammopathy	Type of Ig	Targeted Antigen	Clinical Picture	Refs.
Waldenström’s macroglobulinemia	Macroglobulin	Prothrombin	Hyperprothrombinemia	[38]
Macroglobulinemia	“Fast” gamma-globulins	Cold agglutinin activity	Acquired hemolytic anemia	[39]
Waldenström’s macroglobulinemia	IgM	Rheumatoid factor properties	Cold agglutinin activity	[40]
Multiple myeloma	IgA	Coagulation factor VIII	Bleeding	[41]
Waldenström’s macroglobulinemia	IgM	Immunoglobulin		[42,43]
Waldenström’s macroglobulinemia	IgM	Coagulation factor VIII	Bleeding	[44]
Multiple myeloma		Antibody activity		[45]
Multiple myeloma		Antibody activity		[46]
Waldenström’s macroglobulinemia	IgM-k	IgG	Hyperviscosity syndrome	[47]
Multiple myeloma	IgG	Insulin	Hypoglycemia	[48]
Waldenström’s macroglobulinemia	IgM-k	Myelin	Chronic demyelinating polyneuropathy	[49]
Waldenström’s macroglobulinemia	IgM	Myelin	IgM deposits on the myelin sheath	[50]
Waldenström’s macroglobulinemia	IgM	Nerve antigens	Polyneuropathy	[51]
Waldenström’s macroglobulinemia	IgM	ANA		[52]
Paraproteinemia	IgM-k	Myelin	Polyneuropathy	[53]
Multiple myeloma	IgG	Insulin	Insulin resistance	[54]
MGUS	IgM	Insulin	Insulin resistance	[54]
Waldenström’s macroglobulinemia	IgM	Thyroid hormones T3, T4	Hypothyroidism	[55]
Multiple myeloma	IgA-κ	IgG	Glomerulonephritis	[56].
Waldenström’s macroglobulinemia	IgM	Myelin	Polyneuropathy	[57]
Waldenström’s macroglobulinemia	IgM	Myelin	IgM deposits on the myelin sheath	[58]
Paraproteinemia	IgM	MAG	Demyelinating neuropathy	[59]
MGUS	IgM-κ	GD2, GD3, GD1b, GT1b	Polyneuropathy	[60]
Paraproteinemia	IgA-κ	Apolipoproteins B and E	Immune complexes in retinal blood vessels	[61]
Multiple myeloma	IgG-κ	Insulin	Hypoglycemic seizure	[62]
MGUS, multiple myeloma, Waldenström macroglobulinemia	Paraproteins	ANA, DNA		[63]
MGUS, multiple myeloma, Waldenström macroglobulinemia	Paraproteins	Histone		[64]
Monoclonal gammopathy	Paraproteins	DNA, cardiolipin, RF, ENA		[65]
Paraproteinemia	IgM	GD1a ganglioside	Motor neuropathy	[66]
Multiple myeloma	IgG-λ	Insulin	Hypoglycemic seizure	[67]
Multiple myeloma	Paraproteins	ANA: Sm, RNP, DNA, histones		[68]
Monoclonal gammopathies	IgG, IgM, IgA	ANA: Sm, RNP		[69]
Monoclonal IgM gammopathy	IgM	Gangliosides	Neuropathies	[70]
Waldenström’s macroglobulinemia	IgM-κ	IgG3		[71]
Multiple myeloma	IgG-λ	Insulin	Hypoglycemic seizure	[72]
MGUS	IgM-κ	GT1b, GD1a, GD1b, GM3, GD3	Ataxic polyneuropathy	[73]
MGUS	IgM	GD1b	Sensory neuropathy	[74]
Monoclonal gammopathy	IgG, IgM, IgA	Ro/SS-A, La/SS-B		[75]
Paraproteinemia	IgG	Insulin	Hypoglycemia	[76]
Monoclonal gammopathy	IgG, IgM, IgA	Thyroglobulin		[77]
Multiple myeloma	IgA	T3, T4	Hyperthyroxinemia	[78]
Monoclonal gammopathy	IgM	MAG	Demyelinating neuropathy	[79]
Monoclonal IgM gammopathy	IgM	MAG, SGPG	Polyneuropathy	[80]
Monoclonal gammopathy		C1-inhibitor	Acquired angioedema	[81]
MGUS	IgM	MAG	Peripheral neuropathy	[82]
Paraproteinemia	IgM	Carbohydrate epitopes on glycoproteins and glycolipids	Polyneuropathy	[83]
Waldenström’s macroglobulinemia	IgM	MAG	Neuropathy	[84]
MGUS	IgM	MAG	Peripheral neuropathy	[85]
MGUS	IgG	Tubulin, GD1a, GM1, P0-like myelin glycoprotein, chondroitin sulfate C	Peripheral neuropathy	[86]
Monoclonal IgM gammopathy	IgM	Myelin sheaths, SGPG/SGLPG, MAG	Polyneuropathy	[87]
Waldenström’s macroglobulinemia, multiple myeloma	IgG, IgA, IgM	Dermal-epidermal junction antigen	Subepidermal autoimmune bullous skin diseases	[88]
Paraproteinemia	IgA-κ	GBM	Recurrent Goodpasture’s disease	[89]
MGUS	IgA	Myelin	Polyneuropathy	[90]
Paraproteinemia	IgM	Nerve glycolipid antigens (MAG, gangliosides, glycolipids)	Demyelinating neuropathies	[91]
Monoclonal gammopathy	Paraproteins	C1 inhibitor	Acquired angioedema	[92]
Waldenström’s macroglobulinemia	IgM	Glucoproteins, immunoglobulins, ANA	Immune complexes	[93]
Paraproteinemia	IgG-κ	Insulin	Hypoglycemia	[94]
Paraproteinemia	IgA1-κ	α1/α2 chains of type IV collagen	Recurrent Goodpasture’s disease	[95]
MGUS	IgM	MAG, SGPG, SGLPG	Distal sensorimotor neuropathy	[96]
Monoclonal IgM gammopathy	IgM	SGPG, SGLPG, MAG	Polyneuropathy	[97,98]
Multiple myeloma	IgA-κ	Insulin	Hypoglycemia	[99]
Waldenström’s macroglobulinemia	IgM	Amyloid beta peptide	Antitoxic in neuronal cell cultures	[100]
Monoclonal IgM gammopathy	IgM	MAG	Demyelinating neuropathies	[101]
MGUS and multiple myeloma	IgG3, IgA	Paratarg-7		[102]
MGUS and Waldenström’s macroglobulinemia	IgM	Paratarg-7		[103]
Multiple myeloma	IgG-λ	Insulin	Hypoglycemia	[104]
Multiple myeloma	Paraprotein	T3	Thyrotoxicosis	[105]
Paraproteinemia	IgM	GD1b, GQ1b, GT1a	Chronic ataxic neuropathy	[106]
Monoclonal gammopathy	IgM	GM1, GD1a, GD1b, GM2, GQ1b, MAG	Sensory neuropathy	[107]
MGUS, multiple myeloma, Waldenström’s macroglobulinemia	Paraproteins	Sumoylated heat-shock protein 90 β isoform-α (HSP90-SUMO1)		[108]
Monoclonal gammopathy	IgA-λ	Complement factor H	Distal angiopathy and atypical hemolytic uremic syndrome	[109]
MGUS	Paraproteins	Insulin	Hypoglycemia	[110]
Multiple myeloma	IgG-λ	Insulin	Hypoglycemia	[111]
Multiple myeloma	IgA	Cutaneous antigen	Subepidermal blistering dermatosis	[112]
MGUS, multiple myeloma, Waldenström’s macroglobulinemia	IgG, IgM	von Willebrand factor	Bleeding	[113]
MGUS, smoldering multiple myeloma, multiple myeloma		Glucosylsphingosine		[114]
Monoclonal gammopathy	IgG, IgA, IgM	MAG	Neuropathy	[115]
MGUS, Waldenström’s macroglobulinemia	IgM-κ, IgM-λ	MAG	Neuropathy, glioblastoma	[116]
MGUS	IgG-κ	Insulin	Hypoglycemia	[117]
MGUS		Insulin	Hypoglycemia	[118]
MGUS	IgM-κ	MAG	Demyelinating peripheral neuropathy	[119]
Monoclonal IgM gammopathy	IgM	MAG, SGPG, SGLPG	Polyneuropathy	[120]
MGUS	IgM, IgG, IgA	C1-Inhibitor	Angioedema	[121]
Smoldering multiple myeloma	IgG, IgA	Glucosylsphingosine		[122]
MGUS, Waldenström’s macroglobulinemia	IgG, IgM	von Willebrand factor	Bleeding	[123]

ANA, antinuclear antibody; ENA, extractable nuclear antigens; GBM, Glomerular basement membrane; MAG, myelin-associated glycoprotein; MGUS, monoclonal gammopathy of undetermined significance; SGLPG, sulfated 3-glucuronyl lactosaminyl paragloboside; RF, rheumatoid factor; RNP, ribonucleoprotein; SGPG, sulfated 3-glucuronyl paragloboside.

Some authors claim that for about 20–60% of MGUS and myeloma patients, the target of the monoclonal IgG and IgA was either an Epstein–Barr virus (EBV), herpes simplex virus 1 (HSV-1), varicella zoster virus (VZV), cytomegalovirus (CMV), HCV, *H. pylori*, or a glucolipid glucosylsphingosine (GlcSph), also known as lysoglucosylceramide (LGL1) [124,125]. Interestingly, the use of the multiplex infectious antigen microarray enabled the identification of targets (paratags) for more than 60% of purified monoclonal immunoglobulins [36]. Other data suggest that 11% of serum samples from multiple myeloma patients were anti-HCV-positive [126]. These and other data allowed Bosseboeuf et al. to state that chronic stimulation by infection antigens or autoantigens may lead to the emergence of oligoclonal and eventually, monoclonal plasma cells, producing monoclonal immunoglobulins associated with MGUS, smoldering multiple myeloma, or multiple myeloma [114,125]. In any case, the association between HBV and HCV infections and an increased risk of developing multiple myeloma emphasizes the importance of monitoring patients with chronic hepatitis for the development of hematological malignancies [127].

## 3. Monoclonal Gammopathies and Autoimmune Diseases

Earlier comprehensive literature reviews on autoimmune disorders identified in patients with monoclonal gammopathies have emphasized an increased prevalence of autoimmune conditions in patients with multiple myeloma and MGUS, including various autoimmune hematologic and rheumatologic conditions [128]. Epidemiologic data revealed an increased risk of gammopathy in the setting of autoimmune or allergic conditions [129]. The authors reported a significantly elevated risk of multiple myeloma associated with a broad category of autoimmune disorders and specific prior autoimmune disorders, including polymyositis/dermatomyositis, systemic sclerosis, autoimmune hemolytic anemia, pernicious anemia, and ankylosing spondylitis. Observed strong associations between pernicious anemia and MGUS/multiple myeloma, suggesting that the anemia seen in plasma cell dyscrasias may be of autoimmune origin.

New clinical studies have confirmed the potential association between multiple myeloma and autoimmune diseases. For instance, Jin et al. reported a statistically significant correlation between an increased risk of primary sclerosing cholangitis and multiple myeloma, whereas a negative correlation was observed between multiple myeloma and rheumatoid arthritis [130]. At the same time, no association was found for type 1 diabetes, SLE, psoriasis, multiple sclerosis, primary biliary cirrhosis, and juvenile idiopathic arthritis. Autoimmune diseases with systemic and organ involvement, polymyositis/dermatomyositis, rheumatoid arthritis, systemic sclerosis, pernicious anemia, and ankylosing spondylitis have also been reported to be associated with an increased MGUS risk [131]. A family history of autoimmune disease is associated with a significantly increased risk of MGUS, but not multiple myeloma [132,133,134]. However, in patients with multiple myeloma and a prior autoimmune condition, the risk of death was significantly increased [135]. At the same time, results from another population-based study suggested that the presence of autoimmune conditions was interrelated with a lower risk of evolvement from MGUS to multiple myeloma or other lymphoproliferative diseases [136]. The authors speculated that if MGUS is induced by prolonged antigenic stimulation, it is unlikely to sustain the genetic alterations that may trigger malignant progression. At the same time, a population-based screening study of adults in Iceland found no association between autoimmune disease and MGUS, although an autoimmune reaction was linked to a prior clinical diagnosis of MGUS [137].

Despite some conflicting data on certain autoimmune conditions and PCD, a link between IgA multiple myeloma and rheumatoid arthritis has been repeatedly proposed [138,139]. Additionally, the connection between personal and family histories of Sjögren syndrome and autoimmune hemolytic anemia and the risk of Waldenström’s macroglobulinemia suggests possible shared susceptibility for these disorders [140]. Overall, while many epidemiological studies, case reports, and population-based research show a higher prevalence of MGUS and multiple myeloma among people with a history of autoimmune conditions, some discussions point out methodological limitations in these findings [137,141].

The strongest evidence for a mechanism starting with autoimmunity that leads to monoclonal gammopathy is found in children. Data from a large urban children’s hospital shows that monoclonal gammopathies are very rare in kids under 18, with population studies indicating a prevalence well below 0.1% in this age group. When monoclonal gammopathies are found in children, they are almost always linked to underlying issues like immune deficiencies, genetic syndromes, or chronic illnesses, rather than being incidental findings as seen in adults. Large pediatric studies have found no cases of multiple myeloma or Waldenström macroglobulinemia in children, and monoclonal proteins are usually identified in cases of secondary causes or immune problems rather than classic MGUS [142,143].

Autoimmunity, as an immune phenomenon, is certainly a chicken-and-egg question in cancer, including monoclonal gammopathy-related neoplasms. Importantly, even if a definitive causative role cannot be established in all cases, the connection between immune-mediated reactions or chronic immune stimulation and plasma cell dyscrasia cannot be ignored. Studying the prevalence and distribution of monoclonal gammopathy in patients with autoimmune diseases, as well as the development of autoimmune conditions in patients with monoclonal gammopathy, will help evaluate and understand the possible causal role of persistent antigen stimulation in the pathogenesis of B-cell malignancy (Figure 1). Evidence suggests that autoimmune diseases may be indolent B-cell lymphoproliferative disorders, extending the forbidden clone hypothesis proposed in the 1950s. This hypothesis has gained renewed interest, particularly in hematologic neoplasms, since clonality of B and plasma cells has been demonstrated in some autoimmune diseases, supporting the idea of autoreactivity within the B-cell lineage [11,144]. Could mutations in genes that encode transcription factors early in B-cell development allow autoimmune B-cell clones to persist and proliferate, escaping deletion and producing autoantibodies? Could genetic abnormalities in these escaped autoimmune B-cell clones, such as translocations in the *IGH* gene, rearrangements of the *MYC* oncogene, deletions of 1p, 13q, or 17p, or mutations in *KRAS*, *NRAS*, *BRAF*, or *TP53*, initiate the process of “myelomagenesis,” leading to the development of multiple myeloma and its progression?

Additionally, there may be a shared genetic susceptibility for developing both autoimmune diseases and multiple myeloma/MGUS. Similarly, an autoimmune reaction, although not directly causative, might still play a significant role in the development of hematological neoplasms, especially multiple myeloma, whose progression is driven by immune dysregulation within the tumor microenvironment [145]. Inflammation related to autoimmunity may trigger MGUS and multiple myeloma. B cells in multiple myeloma rely on inflammatory pathways activated by factors such as IL-6, IL-13, TNF-α, B-cell-activating factors, and TLR ligands [146,147,148]. More recently, several regulatory roles have been identified for small non-coding microRNAs (miRNAs), which suppress target gene expression through post-transcriptional pathways. Disturbance of numerous miRNAs has been observed in autoimmunity and autoimmune diseases, with implications for both pathogenesis and therapy [149,150]. Additionally, viral miRNAs were associated with the development of autoimmunity due to their interactions with the host genetic code and resulting genomic instability, as seen with the Epstein–Barr virus [150,151,152]. Simultaneously, several miRNAs are known to regulate pathways involved in multiple myeloma initiation, development, apoptosis, and progression, such as Wnt/β-catenin, PI3K/Akt/mTOR, p53, and KRAS [142,143]. Notably, some B-cell-specific miRNAs that control B-cell tolerance, autoantibody production, B-cell differentiation, and their associated regulatory networks may serve as molecular links connecting autoimmunity and B-cell malignancies [153]. Detailed experiments are necessary to further explore the emerging link between autoimmunity and cancer via dysregulation of specific miRNAs.

## 4. Monoclonal Autoantibody in Plasma Cell Dyscrasias

It is conceivable that unusual clinical consequences may occur when monoclonal myeloma paraproteins bind to host endogenous antigens. Published clinical data well illustrate this possibility. Many patients with multiple myeloma and related plasma-cell dyscrasias have been reported to exhibit clinical manifestations of specific antigen–antibody interactions between a monoclonal immunoglobulin produced by proliferating B-cell or plasma cell clones and autologous antigens. The first documented reports of the functional properties of “M” components, now known as M-proteins or paraproteins, appeared at the end of the 1950s (Table 1). The abbreviation of “M” was introduced by Putnam and Udin, who reported the physicochemical properties of serum proteins in multiple myeloma in their pioneering studies [154]. Since that time, numerous case reports and clinical experimental studies have described and often demonstrated that paraproteins isolated from patients with PCD can recognize and bind to various autoantigens (Table 1). In many cases of MAGa, an autoimmune reaction results in a specific clinical picture that resolves after the autoreactive paraprotein is successfully eliminated or reduced.

### 4.1. Paraproteinemic Autoimmune Insulin Syndrome

Redmon et al. described a patient with a hypoglycemic seizure and the features of the autoimmune insulin syndrome, i.e., insulin-binding autoantibodies [72]. However, the analysis of antibodies revealed that the patient had suffered from multiple myeloma, and his spontaneous hypoglycemia was due to a monoclonal insulin-binding antibody produced and released by malignant cells. The IgG-lambda M-spike was detected by serum protein electrophoresis and immunoelectrophoresis and confirmed by bone marrow biopsy. Interestingly, the monoclonal insulin-binding antibody produced in that patient had a low affinity for insulin but a very high binding capacity [72]. Thus, this clinical case demonstrated that malignant plasma cell clones can produce monoclonal autoantibodies (Table 1). Similarly, Sluiter et al. and Wasada et al. reported monoclonal insulin-binding IgG-κ and IgG-λ autoantibodies in patients with monoclonal gammopathies [62,67]. Many recurrent cases of MGUS and multiple myeloma patients with hypoglycemia, evidently due to IgG or IgA paraprotein targeting insulin, have been published [48,54,76,94,99,104,110,111,117,118]. Many additional cases are discussed in a few reviews [155,156].

### 4.2. Paraproteinemic Autoimmune Neuropathies

The incidence of peripheral neuropathies connected to monoclonal gammopathies is common and well characterized, suggesting that monoclonal paraproteins may have a pathogenetic role in peripheral nervous system injury [53,157,158,159,160]. Approximately 8% of idiopathic neuropathy occurrences are associated with monoclonal gammopathy [19]. Among these cases, IgM accounts for 60%, IgG for 30%, and IgA for 10%. Polyneuropathies associated with IgM paraproteins are the most common peripheral neuropathies among monoclonal gammopathies (Table 1). The role of monoclonal immunoglobulins in the development of peripheral neuropathy has been well established in cases of the IgM monoclonal gammopathy, specifically MGUS or Waldenström’s macroglobulinemia. Peripheral neuropathy can occur in up to 20% of patients with MGUS and most often develops in the context of IgM MGUS [119,161]. Interestingly, the monoclonal antibodies that react with neural antigens are almost always of the IgM class, and peripheral neuropathies connected with IgM MGUS are far more frequent than those linked to Waldenström’s macroglobulinemia [162]. Monoclonal IgM usually demonstrates autoantibody activity to carbohydrate epitopes shared by several nerve-specific antigens. At the same time, IgG or IgA MGUS, as well as the monoclonal component in POEMS (commonly IgA-λ and IgG-λ), have an uncertain causal relationship with the neuropathy [161,162]. Saito stated that the most prevalent antibody directed against myelin-associated glycoprotein (MAG) was IgM, whereas IgG and IgA monoclonal proteins did not react with MAG or other components of peripheral nerve myelin [79]. Furthermore, demyelinating neuropathy associated with IgG- or IgA-MGUS may be indistinguishable from chronic inflammatory demyelinating polyradiculoneuropathy in terms of clinical and electrophysiological features [163]. In general, IgM MGUS and non-IgM MGUS are associated with distinct neuropathies [164].

The comparative analysis of monoclonal IgM from 16 patients with Waldenström’s macroglobulinemia and polyneuropathy and 73 patients with macroglobulinemia without neuropathy revealed shared cross-idiotypic antigenic determinants on the Fab fragments. These were demonstrated by hemagglutination and precipitin reactions in patients with polyneuropathy [51]. The authors concluded that the antibody activity of monoclonal IgM in patients with Waldenström’s macroglobulinemia and polyneuropathy mediates nerve injury by targeting nerve antigens with M-protein or by recognizing factors that contribute to the neuropathy’s pathogenesis. Further analysis of monoclonal IgM from 25 patients with Waldenström’s macroglobulinemia and peripheral neuropathy revealed that 10 cases (40%) exhibited antibody activity against the myelin sheaths [57]. In the remaining patients, the monoclonal IgM, which lacked anti-myelin autoreactivity, displayed either cross-idiotypic antigenic determinants or anti-intermediate-filament autoantibody activity, suggesting heterogeneity among autoreactive monoclonal IgMs in patients with Waldenström’s macroglobulinemia and peripheral neuropathy [57]. These data have been confirmed by other teams [87]. In many patients with IgM MGUS and demyelinating peripheral neuropathy, the presence of anti-(MAG antibodies in the blood has been proven [80,82,85,119]. Knowing that IgM MGUS accounts for 15% of all MGUS cases [165], it is important to mention that similar autoimmune anti-MAG activity has also been observed in a patient with IgA MGUS [90].

Many patients with polyneuropathy and IgM MGUS who are negative for MAG reactivity have monoclonal antibodies that react with gangliosides (Table 1). For instance, Arai et al. observed binding of MGUS IgM-kappa with GT1b, GD1a, GD1b, GM3, and GD3 [73], while Ilyas et al. saw IgM paraprotein reaction with GD2, GD3, GD1b, and GT1b, but not with GM1, GM3, and GD1a [60]. Similar data were reported for patients with MGUS and associated sensory neuropathy [70,74]. A patient with motor neuropathy associated with monoclonal IgM protein targeting GD1a ganglioside was also described [66]. Yuki and Uncini reported that chronic ataxic neuropathy with disialosyl antibodies is associated with IgM paraprotein against GD1b and GQ1b, which sometimes reacts with GT1a [106]. Analysis of 112 cases of neuropathies associated with monoclonal IgM revealed that in 27.5% of cases, monoclonal IgM had no identifiable autoantibody activity [91,166]. However, in 81 cases, monoclonal IgM demonstrated autoantibody activity against nerve glycolipid antigens of the peripheral nerve (72%): myelin sheath in 34,5% of cases, MAG in 38% of cases, glycolipids, sulfate-3-glucuronyl paragloboside (SGPG) and sulfate-3-glucuronyl lactosaminyl paragloboside (SGLPG) in 52% of cases, gangliosides in 21.5% of cases, and sulfatide in 26% of cases [91]. Peripheral neuropathies associated with IgG MGUS demonstrated the presence of paraproteins to one or more neural antigens, including tubulin, P0-like nerve myelin glycoprotein, GD1a, GM1, and chondroitin sulfate C, in 40% of patients with chronic inflammatory demyelinating polyneuropathy-like and 43% with sensory neuropathy, but also in 37% patients with IgG MGUS without peripheral neuropathies [86].

Importantly, the profiles of monoclonal IgM autoantibodies correlated with specific clinical features of different demyelinating neuropathies. Several clinical cases presented monoclonal IgM autoantibodies recognizing SGPG, SGLPG, and MAG antigens, independently or cross-reactively [87,96,97,98]. A comparative analysis of clinical and nerve conduction characteristics in patients with neuropathy associated with IgM monoclonal gammopathy targeting gangliosides or MAG revealed that patients with anti-ganglioside paraproteins could not be differentiated from those with anti-MAG paraproteins [107]. However, among patients with anti-ganglioside antibodies, autoantibodies to GD1b and GQ1b were associated with a purely sensory neuropathy, whereas asymmetric weakness with symmetric sensory loss was associated with anti-asialo-GM1 antibodies [107].

Interestingly, ultrastructural analysis of biopsied peripheral nerve in clinical cases of chronic sensory neuropathy in patients with Waldenström’s macroglobulinemia revealed IgM-positive deposits on the myelin sheath, which appeared to be a contributory factor in the neuropathy [50,58,167]. The pertinent relationship between distal acquired demyelinating sensory neuropathy associated with paraproteins has been reported in CANOMAD (chronic ataxic neuropathy with ophthalmoplegia, M-protein, and anti-disialosyl antibodies) and POEMS syndrome (polyneuropathy, organomegaly, endocrinopathy, M-protein, and skin changes) [163]. Overall, IgM paraproteins account for an unequal 60% of monoclonal gammopathy-related neuropathies [25]. The circulating monoclonal IgM targeting antigens on peripheral nerve carbohydrate structures is commonly associated with MGUS, Waldenstrom’s macroglobulinemia, B-cell lymphoma, or chronic lymphocytic leukemia [166].

### 4.3. Other Types of MAGa

In addition to the well-described paraproteinemic autoimmune disorders discussed above, many patients with plasma-cell dyscrasias have been reported to show clinical manifestations due to specific antigen–antibody interactions. These interactions occur between monoclonal immunoglobulin produced by a proliferating B-cell clone and self-antigens. Many clinical cases have demonstrated detectable autoantibody activity in monoclonal IgM immunoglobulins in patients with Waldenström’s macroglobulinemia (Table 1). For example, Intrator et al. demonstrated strong immunofluorescent staining of unfixed cryostat sections of rat or mouse kidney tissue for IgM paraprotein and its Fab mu fragments isolated from a patient with Waldenström’s macroglobulinemia [52]. Interestingly, the monoclonal IgM with antinuclear activity disappeared after chemotherapy in this patient. Ota et al. reported a Waldenström’s monoclonal IgM-kappa macroglobulin with specific antibody activity against a human IgG (Gm) allotype [71]. Leatham et al. showed that a multiple myeloma IgA paraprotein targets a cutaneous self-antigen, leading to a paraneoplastic recurrent subepidermal blistering dermatosis [112]. The connection between subepidermal autoimmune blistering skin diseases and paraprotein deposition in patients with plasma cell dyscrasias has also been documented [88]. An autoantibody-like IgA-κ paraprotein was isolated from a patient with multiple myeloma and autoimmune hyperlipoproteinemia, which binds to apolipoprotein B-containing lipoproteins [61].

IgM paraprotein in a patient with Waldenström’s macroglobulinemia has been reported to target thyroid hormones T3 (triiodothyronine) and T4 (thyroxine), causing hypothyroidism [55]. The binding of monoclonal immunoglobulins from multiple myeloma to T3 or T4, leading to thyrotoxicosis or hyperthyroxinemia, has been confirmed in other clinical cases [78,105,168]. Larsson et al. described a case of falsely elevated Thyroid-Stimulating Hormone results due to proven interference by a monoclonal IgG-λ paraprotein in multiple myeloma [169].

A monoclonal IgA-κ antibody, identified by serum protein electrophoresis, that recognizes the glomerular basement membrane (GBM) antigen and causes recurrent Goodpasture’s disease has also been described [89]. A similar case has been reported in which an IgA1-κ paraprotein targeting the α1/α2 chains of type IV collagen induced anti-GBM disease that progressed to end-stage renal disease [95]. This is a rare case of recurrent Goodpasture’s disease secondary to monoclonal autoreactive nephritogenic IgA antibodies. Anti-gammaglobulin or -rheumatoid factor activity of monoclonal IgM, IgG, and IgA paraproteins in patients with PCD has also been reported [170,171]. For instance, IgA-κ monoclonal immunoglobulin binding to IgG in a patient with multiple myeloma has been shown to lead to the later occurrence of clinical symptoms associated with vascular purpura, rheumatoid arthritis, or acute membranoproliferative glomerulonephritis [56].

Several diseases and clinical symptoms may also be associated with paraprotein-mediated autoimmune activity (Table 1). Acquired von Willebrand syndrome, a rare bleeding disorder characterized by alterations in von Willebrand factor (VWF), may be caused by various pathological conditions, including lymphoproliferative and myeloproliferative diseases. Well-documented acquired von Willebrand syndrome secondary to monoclonal gammopathy is an important association in adults. The etiological link of several cases of acquired von Willebrand syndrome with monoclonal gammopathy has been repeatedly established when it was reported that M-proteins from IgG MGUS, IgM Waldenström’s macroglobulinemia, and IgG multiple myeloma patients may bind to or interfere with VWF and be responsible for its immunologic clearance in plasma [113,123]. Interestingly, acquired coagulation factor VIII (known as antihemophilic factor A) deficiency in myeloma or associated with the autoimmune paraprotein activity has also been described [41,44].

Interesting cases of acquired angioedema due to C1-inhibitor deficiency, caused by anti-C1-inhibitor antibodies, have been reported by Gobert et al. [172]. The associated diseases were primarily non-Hodgkin lymphoma and MGUS. A few patients had myeloma and amyloid light-chain amyloidosis. Although the autoimmune specificity of paraproteins has not been determined, the identity of the anti-C1-inhibitor isotype with the M-spike isotype was documented for IgG, IgM, and IgA antibodies [172]. Similarly, Frémeaux-Bacchi et al. reported an association between acquired angioedema and monoclonal gammopathy in more than 60% of cases and identified a monoclonal immunoglobulin peak with the same heavy- and light-chain isotypes as the acquired anti-C1 inhibitor antibody [92]. The demonstration of the C1-inhibitor binding ability of monoclonal immunoglobulins in these patients has also been reported [81]. Notably, successful chemotherapy for B-cell malignancies is associated with complete remission of angioedema [173]. A recent analysis of patients with angioedema due to acquired C1-Inhibitor deficiency associated with MGUS also concluded that complete remission of angioedema correlated with the disappearance of monoclonal immunoglobulin in serum, suggesting the remission of the primary hematological abnormalities [121].

Several studies have investigated the association between antinuclear antibodies (ANA) and monoclonal gammopathies, focusing on the ability of paraproteins to exhibit ANA activity. The ANA test is a common blood test used to screen for a wide spectrum of autoantibodies. In 1979, Intrator et al. reported ANA targeting by a monoclonal IgM protein from a patient with Waldenstrom’s macroglobulinemia [52]. Shoenfeld et al. demonstrated ANA, histone, and DNA-binding activity in serum and in isolated monoclonal immunoglobulins in a cohort of patients with plasma cell dyscrasia [63,64]. Analysis of sera from patients with monoclonal macroglobulinemia revealed that ANA targeting was documented in 11% of sera [93]. Similarly, screening of sera from 75 patients with monoclonal gammopathies for various autoreactivity, such as binding to DNA, cardiolipin, extractable nuclear antigens, and rheumatoid factor, revealed that 23% of patients possessed autoreactivity [65]. Numerous confirmatory results have been published over the last few decades: anti-Sm, anti-RNP, anti-histones [68,69], and anti-Ro/SS-A and anti-La/SS-B [75] activities of monoclonal immunoglobulins have been detected.

**Figure 3:**
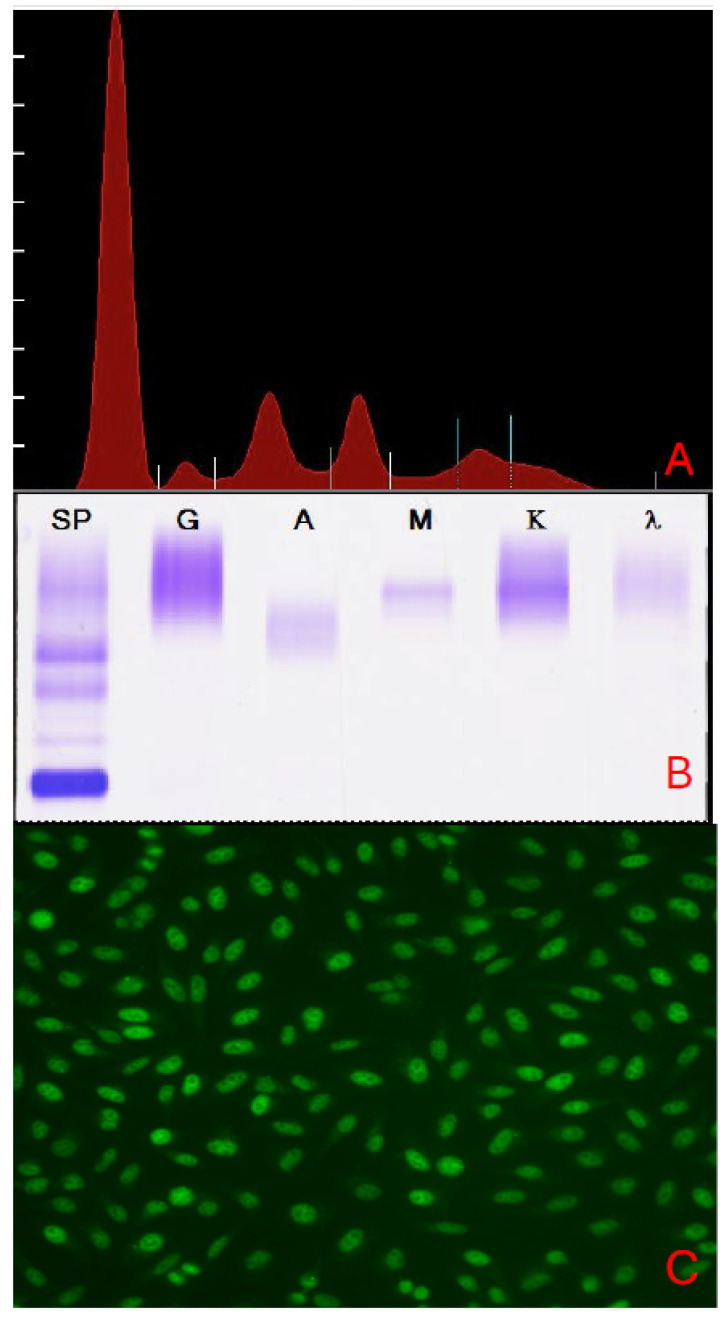
**Example of an antinuclear antibody (ANA)-positive immunoglobulin M (IgM) gammopathy.** Peripheral blood was collected in serum separator tubes, allowed to clot, and centrifuged to obtain serum for analysis. Serum protein electrophoresis (SPEP) and immunofixation electrophoresis (IFE) were performed on agarose gels using standardized laboratory protocols, as described in the legend to Figure 1. Panel (**A**) shows SPEP results of Sample 4 demonstrating a monoclonal spike in the gamma region, consistent with monoclonal gammopathy. The M Spike values in our sampled population ranged from 0.25 to 4.41 g/dL, with both the mean and median of 0.42 g/dL. Panel (**B**) displays IFE results for the same sample, confirming the monoclonal nature and IgM isotype of the paraprotein. Panel (**C**) presents the results of Antinuclear antibody (ANA) testing of Sample 4, performed by indirect immunofluorescence (IFA) on HEp-2 cell substrate, with fluorescence microscopy evaluation according to manufacturer guidelines, showing positive ANA reactivity with a coarse speckled pattern. IFA was performed using the Helios Automated IFA System (AESKU Group, Thermo Fisher Scientific Inc., Portage, MI, USA) according to the manufacturer’s instructions, as described [177]. All tests were performed at the College of American Pathologists-accredited, CLIA-certified UPMC Clinical Immunopathology Laboratory.

**Figure 4:**
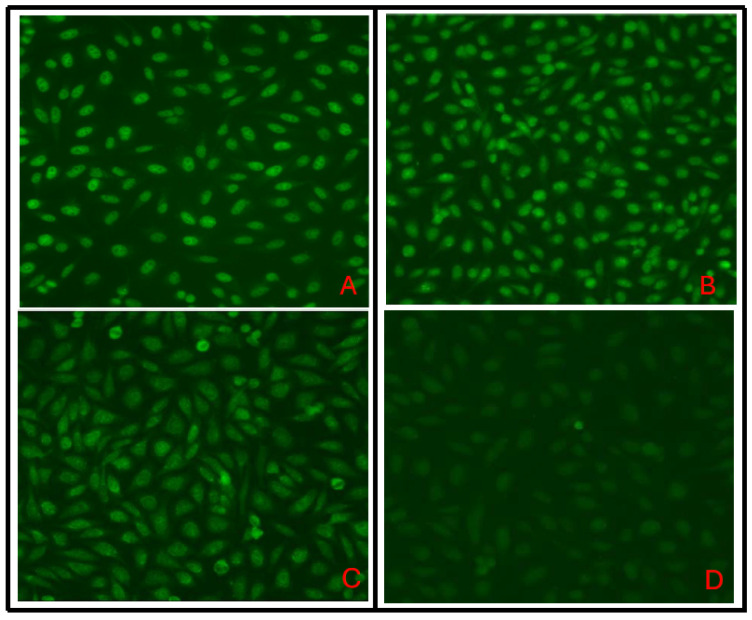
**Spectrum of ANA reactivity within a limited patient cohort.** Antinuclear antibody (ANA) testing was performed on patient remnant serum samples using indirect immunofluorescence on HEp-2 cells, following the manufacturer’s recommended protocols as described in the legend of Figure 2. Serum samples were screened at a 1:80 dilution, and the estimated titer was determined by fluorescent intensity using negative and positive controls with the AESKU software package (Version 3.3). Briefly, slides were incubated with diluted serum samples, washed to remove unbound antibodies, and then incubated with a fluorescein-labeled anti-human IgG conjugate prior to automated fluorescence microscopy, capturing three images from different areas of the slide. ANA patterns and titers were interpreted independently by two pathologists using standardized reporting criteria. Digital pattern resolution images: Panel (**A**) shows Sample 4 demonstrating 1:160 reactivity with a coarse speckled ANA pattern; Panel (**B**) shows Sample 7 demonstrating 1:160 reactivity with a homogeneous ANA pattern; Panel (**C**) shows Sample 6 demonstrating 1:80 reactivity with a speckled ANA pattern; and Panel (**D**) shows Sample 11 demonstrating non-reactive ANA. All tests were performed at the College of American Pathologists-accredited, CLIA-certified UPMC Clinical Immunopathology Laboratory.

In our pilot studies, we randomly collected 34 serum samples with confirmed monoclonal IgM immunoglobulins, as determined by serum protein electrophoresis and immunofixation (Figure 2): the mean ± SEM M-spike size was 1.11 ± 0.17, median 0.82, range 0.23–4.41 g/dL. Of these, 29 (85%) were IgM-κ (Table S1). All samples were tested using an ANA screening assay described in Figure 3. The results revealed that 13 samples (38%) were ANA-reactive, with titers ranging from 1:80 to 1:1280. ANA staining patterns varied as well, with the homogenous pattern being the most common (38%) and the speckled pattern the second most common (31%) (Table S1). Additional ANA patterns included centromere, rods and rings, and the GW bodies. The average titer of the homogenous pattern was higher than in the speckled pattern. The centromere ANA pattern demonstrated the higher autoantibody titer (Table S1). Interestingly, in the general population, the speckled pattern is the most common and is associated with autoantibodies targeting proteins involved in RNA processing. Representative results of ANA patterns seen in our study are shown in Figure 4. It is important to note that monoclonal IgM immunoglobulins were not isolated from the patients’ sera; therefore, autoreactivity cannot be directly linked to paraproteins without further analysis. Interestingly, two ANA-positive samples were also tested for 14 common autoantigens using a multiplexed ANA screen assay on the BioPlex 2200 (Bio-Rad Laboratories, Inc., Hercules, CA, USA) according to standard clinical diagnostic procedures. Both samples tested negative for all individual analytes. Despite the limitations of a small pilot study sample and the focus solely on IgM monoclonal immunoglobulins, the results support the MAGa concept and lay a strong foundation for a comprehensive analysis of the autoimmune properties of monoclonal immunoglobulins in patients with PCD.

Thus, numerous data demonstrate that monoclonal immunoglobulins in patients with PCD may target self-antigens. For instance, one comprehensive comparative study reported that 71% of serum samples harvested from monoclonal macroglobulinemia patients bound autoantigens, and that some of the resulting immune complexes led to clinically significant manifestations [93]. The authors concluded that in many cases, monoclonal immunoglobulins may be functional autoantibodies rather than “paraproteins.” Patients with Waldenstrom’s macroglobulinemia, for instance, with MAGa, often present with common self-antigen-targeted reactions, including peripheral neuropathy, mixed cryoglobulinemia, or hemolytic anemia at an earlier stage if compared with patients without obvious autoantibody activity [174,175]. The manifestation of autoreactive monoclonal immunoglobulins thus affects the medical history and clinical appearance of the disorder. A depiction of antigen-recognition function may provide important insights into the pathogenesis of monoclonal gammopathies and potentially slow their development and progression. Further development of autoantigenomics, utilizing modern, specific technological and AI approaches [176], should accelerate understanding of the clinical and therapeutic significance of the MAGa concept.

## 5. Mechanisms of MAGa Pathogenesis

A key unanswered question regarding the spectrum of targeted autoantigens in patients with various types of monoclonal gammopathies is the somewhat limited list of identified and verified antigenic targets. The neuropathies associated with monoclonal IgM gammopathy are known to react with glycoconjugated antigenic epitopes on sulfated glucuronic glycolipids, including SGPG and SGLPG, MAG, and sulfatide. Occasionally, monoclonal IgM targets various gangliosides [97]. At the same time, a list of neural autoantigens established for autoimmune neuropathies and paraneoplastic syndrome is significantly bigger than that known for MAGa. However, it is essential to recognize that autoantigens associated with monoclonal IgM gammopathy are limited and specific for peripheral nerve antigens. Almost 50% of patients with monoclonal IgM-associated distal, sensory, or sensory–motor demyelinating polyneuropathies demonstrate serum antibodies to MAG and SGPG, which recognize the HNK-1 carbohydrate epitope [80,178]. It is also possible that monoclonal autoimmune responses to different autoantigens might utilize slightly different immunological pathways. For instance, a reduced neuropathy treatment response has been shown in patients with IgM paraproteins who developed neuropathies in the absence of MAG-targeting antibodies [120].

Beyond their role in clinical manifestations, the antigenic targets of MGUS and multiple myeloma paraproteins may contribute to the pathogenesis of these neoplasms, as was speculated several decades ago [45,46]. Chronic stimulation by these autoantigens may contribute to malignant transformation: chronic autoantigen stimulation induces chronic inflammation, immune dysregulation, and clonal B-cell expansion, all of which can support the development of B-cell malignancies (Figure 1). For example, sequence studies of VH genes encoding clonal immunoglobulins in malignant plasma cells revealed a common pattern of extensive somatic hypermutation and a lack of intraclonal variation [179]. This may suggest that the cancerous cell arises at a mature, post-follicular stage of B-cell development, consistent with a prior role for antigen in selecting the B-cell of origin. However, the antigens that may help reveal the origins of most MGUS and myeloma clones are not yet elucidated. Some data suggest that the hyperphosphorylated modification of the stomatin-like 2 protein (also known as HSPC108 or paratarg-7) may serve as a target for certain paraproteins and be an inherited risk factor for the development of gammopathies [102,180]. Of note, more than 15% of IgG and IgA paraproteins from almost 200 serum samples from patients with MGUS and multiple myeloma recognized paratarg-7 (paraprotein target) [181]. These results were confirmed by analyzing samples from patients with IgM MGUS and Waldenström’s macroglobulinemia [103,180].

Furthermore, evidence of somatic immunoglobulin gene mutations in Waldenström’s macroglobulinemia also suggests a role for antigenic stimulation in the development of this malignancy [13,103,182]. These results also suggest a causal relationship between MGUS, Waldenström’s macroglobulinemia, and chronic antigenic stimulation. For instance, Hermouet et al. reported a case of HCV infection followed by plasma-cell leukemia, in which blast cells were infected with HCV, and their monoclonal immunoglobulin recognized the HCV core protein [183]. It was also suggested that paraproteins in patients with PCD and HCV are directed against the virus [35]. It was also estimated that approximately 10% of HCV-infected patients may develop monoclonal immunoglobulins [184,185,186].

The prevalence of monoclonal gammopathies and malignancies in Gaucher disease, an inherited, lysosomal storage disease, has been repeatedly reported [187]. The accumulation of glucosylceramide and glucosylsphingosine (GlcSph) due to a hereditary deficiency of glucocerebrosidase, along with associated chronic lipid-mediated metabolic inflammation in Gaucher disease, may increase the risk of monoclonal gammopathy. This is because GlcSph may serve as a mediator of B-cell stimulation and as an antigenic target for Gaucher disease-associated PCD [35]. The reactivity of clonal immunoglobulin in Gaucher disease to GlcSph has also been demonstrated. Furthermore, monoclonal paraproteins detected in patients with Gaucher disease and MGUS or multiple myeloma have been reported to specifically target glycosphingolipid antigens presented by macrophages via CD1d, a non-classical MHC class I-related protein [188,189]. These findings support the hypothesis that links Gaucher disease-associated gammopathy to somatic mutations driven by chronic immune activation: prolonged immune activation by lysolipids, lyso-glucosylceramide, and lyso-phosphatidylcholine, may trigger both Gaucher’s disease-associated gammopathies and some sporadic monoclonal gammopathies, as clonal immunoglobulin in 33% of sporadic human monoclonal gammopathies is also specific for lyso-glucosylceramide [188]. Thus, antigenic stimulation may mediate a first polyclonal stage, followed by the evolution of monoclonal tumors enriched in nonhyperdiploid genomes, which are responsive to the triggering antigen [190]. The key conclusion from this model is that targeting the original causative antigens may prevent the progression of premalignant diseases to clinical multiple myeloma.

Another study confirmed that post-translationally modified proteins may serve as autoimmunogenic targets and are molecularly defined risk factors for monoclonal gammopathy. The results revealed the presence of a specific isoform-α of sumoylated heat-shock protein (HSP) 90β (HSP90-SUMO1) in all tested patients who demonstrated paraproteins that target HSP90-SUMO1 [108]. The appearance of HSP90-SUMO1 as a paraprotein target was explained by an autosomal-dominant mutation in the SUMO peptidase sentrin/SUMO-specific protease 2, resulting in deficient desumoylation of HSP90-SUMO1. Importantly, only patients with MGUS, multiple myeloma, and Waldenstrom’s macroglobulinemia who carry the specific isoform of HSP90-SUMO1 were shown to develop paraproteins that target HSP90-SUMO1. This suggests a role for chronic antigenic stimulation in the pathogenesis of these MAGa.

Finally, the repeatedly reported comorbidity between autoimmune diseases and PCD also supports the concept of chronic immune stimulation as a risk factor for B-cell and plasma cell malignant transformation once a specific pattern of mutations emerges or accumulates. Systemic inflammatory rheumatic diseases, including rheumatoid arthritis, SLE, Sjogren’s syndrome, vasculitis, dermatomyositis, or scleroderma, may increase the risk of malignancy, particularly for lymphoproliferative diseases [191,192,193,194]. The chronic inflammatory reaction appears to be the prime risk factor for site-specific cancer and hematologic malignancies [195]. For instance, studies indicate that hematological cancer, mostly lymphoma, is a significant complication of Sjögren’s syndrome [191,196,197,198]. The risk of hematological cancer has been reported to be increased in patients with antineutrophil cytoplasmic antibody (ANCA)-associated vasculitis [199,200,201]. Recent data suggest that patients with SLE have an increased risk for developing hematologic malignancies, while patients with Sjögren’s syndrome or rheumatoid arthritis demonstrate a markedly higher chance of getting non-Hodgkin lymphoma [202].

Thus, the interplay between cancer and autoimmune diseases is complex and is represented by malignancy seen as a comorbidity in rheumatic diseases on one side and tumor-associated rheumatic diseases on the other side. Both associations are characterized by complex sets of conditions that can result from specific pathophysiological mechanisms or arise as a consequence of disease treatment. All cases of self-antigen recognition by paraproteins, monoclonal autoimmune gammopathies, or MAGa, are represented by complicated clinical manifestations and pathological features, frequently rendering them challenging to analyze and affecting patients’ morbidity. Despite this, they remain poorly understood and under-identified.

## 6. Conclusions

There is evidence of increased autoantibody levels in monoclonal gammopathies, which may predict the onset of overt disease. At the same time, many studies, including our own data, highlight the importance of independent analysis of clonal and non-clonal immunoglobulins. Regardless of the autoimmune activity of paraproteins or other non-malignant clonal autoantibodies in patients with PCD, autoimmune complications may significantly impact the prognosis and therapy of monoclonal gammopathies. This involves both the disease’s clinical severity, which is potentially life-threatening, and the disease’s chronic/relapsing clinical course. For example, recent data analyzing ANA positivity and survival in multiple myeloma patients revealed that anti-Sjögren’s syndrome-related antigen A (SSA)-positive patients exhibited significantly shorter progression-free survival than SSA-negative patients [203]. This unusual association of SSA positivity with a higher risk of disease progression in multiple myeloma patients may suggest its potential feasibility as a helpful prognostic marker.

Continuous autoantigen stimulation contributes to the pathogenesis and development of multiple myeloma and other monoclonal gammopathies, underscoring the need for improved awareness, screening, and management. The detection of autoantibody targets is important for diagnosing and managing patients. For instance, multiple myeloma patients who presented with anti-GlcSph paraprotein seemed to have a mild form of the disease, whereas the presence of anti-EBV monoclonal immunoglobulin is associated with severe multiple myeloma [36].

Despite numerous cases observing autoimmune phenomena in patients with MGUS, Waldenstrom’s macroglobulinemia, and multiple myeloma, the persistence or conversion of MGUS paraproteins to autoimmune paraproteins during progression to multiple myeloma, for instance, has not been evaluated. Because PCD encompasses a conceptually distinct spectrum of diseases, reliance on registry-based diagnoses in some studies may introduce misclassification and surveillance bias. In addition, many of the data discussed above are clinical cases, and many do not specify the type of PCD, mentioning paraproteinemia or monoclonal gammopathy. Many cases did not use isolated paraproteins and tested the autoimmune activity of serum samples. Therefore, additional clinical studies, experimental research, and comprehensive epidemiological analysis are needed to determine how to interpret unclear or unproven data related to the MAGa phenomenon.

Furthermore, therapeutic approaches to monoclonal gammopathy, such as hematopoietic stem cell transplantation, CAR T cells, checkpoint inhibitors, and additional monoclonal or bispecific antibodies, may also trigger autoimmune reactions. For instance, the utilization of anti-PD1/PD1L therapy should be carefully considered in patients with paraproteins targeting self-antigens. Thus, the decision to use certain types of therapies in patients with PCD should take into account the disease severity, expectations for disease control, comorbidities, and host and environmental risk factors for severe autoimmune reactions.

Next, identifying and characterizing paratargs (paraproteins’ targets) should improve the efficacy of PCD therapy and potentially reduce the risk of their appearance. For example, a case of ‘targeted antigen reduction therapy’ has demonstrated a reduction in monoclonal immunoglobulins or an improved response to chemotherapy in MGUS and multiple myeloma patients whose paraproteins recognized HCV antigens and were successfully treated with antiviral therapy [204,205]. It is also possible that reducing paratargets may decrease the risk of MGUS transformation and thus prevent the development of multiple myeloma. In fact, in patients with Gaucher disease type 1 and PCD with paraprotein recognizing glucosylsphingosine, which is exclusively elevated in Gaucher disease, GlcSph-reduction therapy caused downregulation of monoclonal immunoglobulins [206]. Conversely, treating the PCD may be associated with remission of specific autoimmune reactions. Complete remission of acquired angioedema in patients with C1-inhibitor deficiency due to MGUS paraprotein is correlated with the complete remission of monoclonal gammopathy, as proven by an undetectable serum M-spike [121]. A better understanding of the complex interplay between myeloma cells and their immune environment should pave the way for the design of more effective immunotherapies with the potential for very long-term disease control.

**Figure 5:**
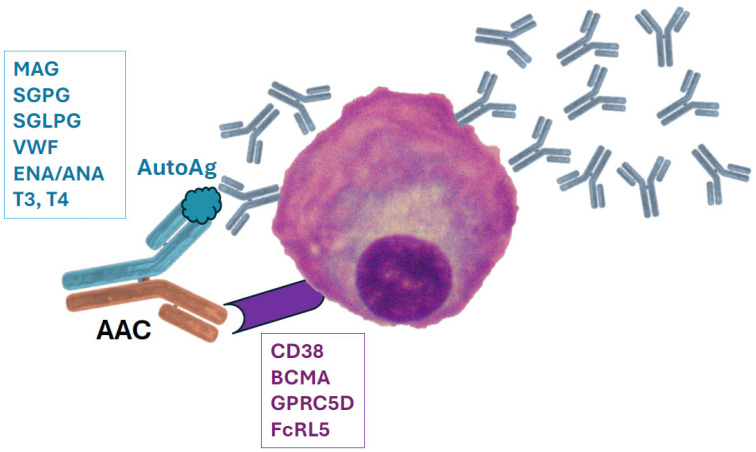
**Autoantigen as an attractive combination partner for CD38-based bispecific antibody construct.** An antibody–antigen conjugate (AAC) targeting CD38 or other markers on plasma cells and delivering an autoantigen payload will selectively accumulate near malignant clones, which are surrounded by monoclonal immunoglobulins recognizing the conjugated autoantigen. This method should enable the elimination of only antigen-specific MAGa plasma cell clones that produce antibodies against known autoantigens. Most non-cancerous plasma cells should remain intact and alive. Ag, antigen(s); ANA, antinuclear antigens; BCMA, B-cell maturation antigen; ENA, extractable nuclear antigens; FcRL5, Fc receptor-like 5; GPRC5D, G protein-coupled receptor, class C, group 5, member D; T3, triiodothyronine; T4, thyroxine; VWF, von Willebrand factor.

The generation of bi-specific antibodies conjugated with a targeted antigen, or antibody–antigen conjugate (AAC), may provide a new approach to targeting multiple myeloma in MAGa. AAC recognizing CD38 or other markers on plasma cells and covalently attached to an autoantigen payload may be selectively accumulated around malignant clones that express and release monoclonal immunoglobulins recognizing the conjugated autoantigen (Figure 5). This approach may increase the cytotoxic AAC-mediated effect on preferentially antigen-specific MAGa plasma cell clones that produce antibodies against known autoantigens. Other non-malignant plasma cells should mainly remain unaffected. This therapeutic approach is still a working hypothesis that requires experimental verification and preclinical validation. The general technique of AAC preparation is well established, and pre-clinical and clinical verification of lower toxicity, higher anticancer activity, and improved stability of AAC recognizing multiple myeloma markers and delivering specific autoantigens may represent a significant trend in the upcoming years.

Another potential implication of MAGa is an increased risk of additional cancers. This phenomenon has recently been reported in patients with glioblastoma who developed classical IgM paraproteinemic neuropathy [116]. Both malignancies are extremely rare, and the chance of random co-occurrence is less than one in a million. In addition to their ability to affect blood–brain barrier permeability and the immune microenvironment within the central nervous system, chronic activation of glial cells by circulating high-titer monoclonal anti-MAG antibody may act as a tumor growth factor, inducing their release from growth inhibition [116].

Finally, one study provides an alternative view on the importance of understanding the MAGa phenomenon and its additional clinical feasibility. To test the hypothesis that monoclonal autoantibodies may have functional activity, IgM antibodies were isolated from the serum of a patient with Waldenström’s macroglobulinemia. These serum monoclonal antibodies demonstrated the CNS binding activity and induced myelin repair in demyelinated lesions by resident oligodendrocytes in an animal model [207]. The amino acid sequences of these antibodies were then determined and used to generate a cDNA library of heavy- and light-chain V regions [208]. These data were used to prepare a recombinant monoclonal antibody that could target oligodendrocytes in situ and in vitro and stimulate remyelination in an animal model of demyelinating disease [209,210]. Similarly, monoclonal IgM antibodies that bind to and stimulate the destruction of β-amyloid have also been isolated from a patient with Waldenström macroglobulinemia [100]. Together, these findings not only provide additional evidence that monoclonal antibodies in patients with monoclonal gammopathies might recognize various host self-antigens but also open the door to unexpected therapeutic uses of MAGa-associated antibodies.

## Data Availability

Due to the nature of this research, participants did not consent to the public sharing of their data, so supporting data are not available.

## Supplementary Materials

The following supporting information can be downloaded at: https://www.mdpi.com/article/10.3390/cancers18111770/s1, Table S1. A single institution’s findings on autoreactive antibodies in patients with plasma-cell dyscrasias, a random selection.

## Abbreviations

AAC	antibody–antigen conjugate
CLL	chronic lymphocytic leukemia
CMML	chronic myelomonocytic leukemia
CMV	cytomegalovirus
EBV	Epstein–Barr virus
FLC	free light chain
GBM	glomerular basement membrane
GlcSph	glucosylsphingosine
HBV	hepatitis B virus
HCV	hepatitis C virus
HSV 1	herpes simplex virus 1
ITP	immune thrombocytopenia
LGL1	lysoglucosylceramide
LPL	lymphoplasmacytic lymphoma
MAGa	monoclonal autoimmune gammopathy
MDS	myelodysplastic syndromes
MGCS	monoclonal gammopathy of clinical significance
MGUS	monoclonal gammopathy of undetermined significance
MZL	marginal zone lymphoma
PCD	plasma cell disorders, plasma cell dyscrasias
PRCA	pure red cell aplasia
RF	rheumatoid factor
SLE	systemic lupus erythematosus
VWF	von Willebrand factor
VZV	varicella zoster virus
