# Supplementary material for: MAGa: Monoclonal Autoimmune Gammopathies
Source: Cancers (Basel). 2026 May 28;18(11):1770. doi: 10.3390/cancers18111770 (PMC13255662; doi:10.3390/cancers18111770)
Supplement: Supplementary file 1 [file cancers-18-01770-s001.zip › cancers-4307822-supplementary.pdf]

Supplementary materials

Table S1. A single institution's findings on autoreactive antibodies in patients with plasma-cell dyscrasias, a random selection.

Sample number	Immunofixation	M Spike (g/dL)	ANA (titer/pattern)
1	M/κ	1.10	Non-Reactive
2	M/κ	1.32	Non-Reactive
3	M/κ	0.25	Reactive 1:80 /Nuclear speckled
4	M/κ	0.42	Reactive 1:160 /Coarse, large speckled
5	M/κ	2.91	Non-Reactive
6	M/κ	0.39	Reactive 1:80/Speckled
7	M/λ	N/A	Reactive 1:160/Homogenous
8	M/κ	2.84	Non-Reactive
9	M/κ	0.83	Non-Reactive
10	M/κ	0.87	Reactive 1:80/Homogenous
11	M/κ	0.62	Non-Reactive
12	M/κ	1.24	Reactive 1:160/Homogenous
13	M/κ	N/A	Non-Reactive
14	M/κ	0.70	Reactive 1:80/Speckled
15	M/κ	0.31,0.42	Non-Reactive
16	M/κ	2.11	Non-Reactive
17	M/κ	0.52	Non-Reactive
18	M/κ	0.23	Non-Reactive
19	M/κ	0.36	Reactive 1:160/GW Body

20	M/κ	0.79	Non-Reactive
21	M/κ	1.25	Reactive 1:160/Rods and Rings
22	M/κ	0.62	Reactive 1:1280/Centromere
23	M/κ	1.57	Non-Reactive
24	M/κ	0.82	Non-Reactive
25	M/λ	1.12	Non-Reactive
26	M/κ	0.52	Non-Reactive
27	M/κ	0.94	Non-Reactive
28	M/κ	1.07	Non-Reactive
29	M/κ	0.52	Non-Reactive
30	M/κ	4.41	Reactive 1:640/Homogenous
31	M/κ	0.60	Reactive 1:320/Homogenous
32	M/κ	3.22	Non-Reactive
33	M/λ	0.27	Reactive 1:80/Homogenous
34	M/λ	1.52	Non-Reactive
